# Gravity Signaling in Flowering Plant Roots

**DOI:** 10.3390/plants9101290

**Published:** 2020-09-29

**Authors:** Shih-Heng Su, Marie A. Keith, Patrick H. Masson

**Affiliations:** Laboratory of Genetics, University of Wisconsin-Madison, 425G Henry Mall, Madison, WI 53706, USA; ssu1@wisc.edu (S.-H.S.); makeith@wisc.edu (M.A.K.)

**Keywords:** flowering plant, primary root, lateral root, gravitropism, auxin, statolith, statocyte, elongation zone

## Abstract

Roots typically grow downward into the soil where they anchor the plant and take up water and nutrients necessary for plant growth and development. While the primary roots usually grow vertically downward, laterals often follow a gravity set point angle that allows them to explore the surrounding environment. These responses can be modified by developmental and environmental cues. This review discusses the molecular mechanisms that govern root gravitropism in flowering plant roots. In this system, the primary site of gravity sensing within the root cap is physically separated from the site of curvature response at the elongation zone. Gravity sensing involves the sedimentation of starch-filled plastids (statoliths) within the columella cells of the root cap (the statocytes), which triggers a relocalization of plasma membrane-associated PIN auxin efflux facilitators to the lower side of the cell. This process is associated with the recruitment of RLD regulators of vesicular trafficking to the lower membrane by LAZY proteins. PIN relocalization leads to the formation of a lateral gradient of auxin across the root cap. Upon transmission to the elongation zone, this auxin gradient triggers a downward curvature. We review the molecular mechanisms that control this process in primary roots and discuss recent insights into the regulation of oblique growth in lateral roots and its impact on root-system architecture, soil exploration and plant adaptation to stressful environments.

## 1. Introduction

As sessile organisms, plants have to use directional cues within their environment to grow towards areas that allow them to best perform their primary functions, which include gas exchange, photosynthesis, and reproduction for shoots, and anchorage, water and nutrients uptake for roots. Examples of such cues include gravity, direction of incident light, gradients in water, ions, oxygen, chemicals, and temperature, and impedance to growth, to cite only a few.

Gravity is a uniform directional cue on Earth, and acquisition by higher plant roots of rapid mechanisms allowing its detection and use as a growth guide contributed to their successful colonization of land ecosystems [1]. Most higher plant organs are capable of using gravity as growth guide, although distinct organs will interpret this signal differently. For instance, primary stems often grow vertically upward, undergoing negative orthogravitropism, whereas primary roots tend to grow vertically downward (positive orthogravitropism). Lateral organs, on the other hand, will often emerge from the primary organs at a stereotypical angle, and progressively change their direction of growth, tending to approach a defined and specific angle from the vertical named gravity set-point angle (GSA). This plagiogravitropic response allows better exploration of the environment surrounding the plant. Leaf petioles and floral pedicels also undergo gravitropism.

A plant organ’s GSA is typically dictated by the genetic makeup of the plant and influenced by environmental and/or developmental parameters. For instance, the GSA of lateral roots conditions the radial expansion of the root system. Environmental parameters will often modify this expansion. For instance, drought will typically dictate a narrower root system that is conditioned by a lower lateral root GSA, leading to deeper root systems capable of seeking out water deep underground. On the other hand, accumulation of nutrients such as phosphorus in upper layers of the soil, will promote an increase in lateral root GSA leading to a radial expansion of the root system and allowing better exploration of the upper layers of the soil. These amazing root-system behaviors are important in agriculture as they allow plants to better adapt to the rapid changes that typically occur in their environments, taking maximal advantage of the resources available to them in close proximity.

This brief and necessarily incomplete description of plant gravitropic responses illustrates the multiplicity of plant organ responses to this directional cue, emphasizing the importance of developmental and/or environmental parameters in guiding organ interpretation of the gravity signal.

To grow, plant organs use a combination of cell division in apical meristems and cell elongation in their elongation zone (EZ). When reoriented within the gravity field, these organs curve back toward their original GSA in a process that involves differential cellular elongation between upper and lower flanks of the EZ. This occurs because plant organs have the ability to sense gravity, transduce the corresponding signal into a biochemical gradient, transmit that gradient to the EZ, and respond by differential cellular elongation between upper and lower flanks of the root. The main objective of this review is to discuss our current understanding of the molecular mechanisms that allow roots to sense the direction of gravity. We also briefly summarize our understanding of the mechanisms that govern signal transmission to the EZ and curvature response.

## 2. Gravity Sensing in Roots

### 2.1. The Columella Cells Contribute to Root Gravity Sensing

In roots, gravity sensing occurs primarily in the cap, an organ that covers the root apical meristem. The root cap serves both as a sensor for several environmental cues and as a protective structure preventing meristem damage as the root grows in soil. Several experiments have documented a role for the root cap in gravity sensing. For instance, deleting the root cap [2], altering groups of cap cells with heavy-ion microbeam irradiation [3], or killing the cap by genetic ablation [4], all lead to defects in root gravitropism. In fact, ablating distinct layers of central columella cells in the cap of *Arabidopsis thaliana* roots demonstrated a predominant role for the top two layers in gravity sensing [5]. So, what makes those cells capable of gravity sensing?

A possible answer to this question can be found in the unique organization of these cells. Indeed, root-cap columella cells are rather large and characterized by a central cytoplasm mostly devoid of visible organelles or bundled cytoskeleton elements. The endoplasmic reticulum (ER) and most other organelles line their periphery, with the exception of dense starch-filled plastids (amyloplasts), which are free to sediment to the bottom under the influence of gravity [6,7,8]. When a plant is reoriented within the gravity field, columella amyloplasts settle to the new bottom-side of the cells, triggering a transduction pathway that leads to altered cellular polarization with subsequent lateral auxin transport, as described below [9].

Several lines of evidence support a role for amyloplast sedimentation and/or repositioning within these cells in gravity sensing. First, studies with starchless and starch-deficient mutants have revealed an important role for amyloplast sedimentation in gravitropism. Starch is denser than the surrounding cytoplasm, providing enough weight for amyloplasts to settle to the lower side of the columella cells. Upon plant reorientation, amyloplasts settle to the new bottom side in wild type plants. On the other hand, in starchless mutants, columella amyloplasts cannot sediment upon gravistimulation, being much lighter. Interestingly, these starchless mutants display substantially altered gravitropic responses, supporting a role for amyloplast sedimentation in gravity sensing [10,11,12,13].

In starch-deficient mutants, a limited amount of starch remains present in the amyloplasts. Yet, the density of these plastids remains insufficient for sedimentation under 1g and root gravitropism is affected. Interestingly, researchers were able to restore a significant level of gravitropic response by subjecting these seedlings to centrifugation, a process that allowed significant sedimentation of starch-deficient plastids [14]. On the other hand, mutations that affect starch degradation (such as *sex1* in *Arabidopsis thaliana*), or conditions that result in larger amyloplasts, all confer greater sensitivity to gravity [15].

Equally significant in demonstrating a role for amyloplast displacement/repositioning in gravity signal transduction are experiments involving strong magnetic fields to artificially displace amyloplasts within the columella cells of the root cap. Such displacement is possible because starch is a diamagnetic material susceptible to the ponderomotive force exerted by local high-gradient magnetic fields. When such magnetic fields are applied near plant organs, directed laterally, they promote amyloplast displacement within the statocytes. In vertical roots, lateral magnetophoretic displacement of statocyte amyloplasts led to root-tip curvature in a direction predicted by amyloplast displacement, which is opposite to that induced in shoots [16,17].

The experiments described above support a role for amyloplast sedimentation in gravisensing. However, it is also important to realize statocyte amyloplasts are not completely free to sediment. In fact, a dynamic actin-filament network appears to also interact transiently with the amyloplasts in these cells. These transient interactions result in saltatory movements that may contribute to or fine-tune the gravitropic response [6,18]. Consistent with this interpretation, treating plants with agents that destabilize actin filaments resulted in enhanced kinetics of gravitropism, increased gravisensitivity and gravitropic-signal persistence leading to overshooting the vertical at the end of a response [19,20]. Similarly, mutations that affect actin dynamics, such as *distorted1*, lead to slower kinetics of gravitropism [18]. Unfortunately, recent reports have also demonstrated a role for the actin-myosin system in modulating the auto-straightening response, a process that allows plant organs to straighten up after curving in response to a tropic stimulus [21,22]. Alterations of the auto-straightening response are also likely to result in the overshooting of a gravitropic response. Therefore, it is currently difficult to determine whether the actin-myosin network truly contributes to the modulation of gravity sensing rather than, or in addition to, also regulating the auto-straightening response.

While amyloplast sedimentation/repositioning in the root-cap statocytes appears to be the primary mechanism of gravity sensing in roots, a secondary mechanism may also exist in the distal elongation zone [23]. To investigate this question, Wolverton and his colleagues modified a microscope to carry a rotating platform in front of its objective. This platform carries a Petri dish that contains a growing seedling, and it is equipped with a motor that automatically rotates the plate to maintain a predefined small segment of the root tip at a specific angle from the vertical. If the system (named the *rotato*) is set up to maintain the root cap at a defined angle from the vertical over time, the platform will rotate continuously as the root curves, attempting to return the tip to the vertical. If, on the other hand, the *rotato* is programmed to maintain a subapical region of the root tip at a defined angle from the vertical, the root should curve until it reaches the vertical, at which time the curvature should end, and the platform should stop its rotation. When this experiment was carried out, the tip continued to curve and the platform continued to rotate, overshooting the vertical [24]. This amazing result suggested cells within the root distal EZ (DEZ) may also be able to sense gravity. In fact, other observations seem to corroborate this conclusion. For instance, decapped maize seedling roots remain somewhat gravitropic, a response that can be enhanced by disrupting actin filaments or manipulating myosin activity [25].

What is very puzzling about these observations is none of the root DEZ cells contain sedimenting amyloplasts, suggesting the existence of an alternative mechanism of gravity sensing in these cells. One possibility would be DEZ cells can sense gravity by detecting the overall pressure exerted by the protoplast on its wall (*hydrostatic pressure model*). A similar mechanism was previously suggested to explain gravity sensing by the large internodal cells of Chara and rice root tips [26,27]. Graviresistance may also contribute to this secondary response. Supported by experiments under microgravity, fractional gravity, and hypergravity conditions [28,29], this phenomenon relates to the ability of plant cells to sense the gravitational pressure through activation of mechanosensitive ion channels, thereby altering the organization of their cytoskeleton network with consequent impact on anisotropic cell expansion and cell wall extensibility [28].

### 2.2. Gravity Sensing in the Statocytes Involves Unknown Receptors

Several models have been proposed to describe the transduction of information derived from amyloplast sedimentation/repositioning within the statocytes into lateral auxin transport, which ultimately leads to a curvature response. The first model suggests the statocytes sense the pressure exerted by sedimenting amyloplasts on sensitive cellular elements (the statolith-pressure model), triggering the opening of mechano-sensitive ion channels [30]. A variant to this model (*tensegrity model*) postulates sedimenting amyloplasts within the statocytes might interact with the cytoskeleton network, thereby disrupting the distribution of tensions within the cell. Connections between actin filaments and the plasma membrane and cell wall would allow force transfer to the membrane, promoting the opening of mechanosensitive ion channels and initiation of gravity signal transduction [31].

These models suggest a role for mechanosensitive ion channels, possibly selective for Ca^2+^, in gravity sensing. Although none of the plant mechanosensitive ion channels identified so far have been shown to contribute to gravitropism [32,33], MCA1 and MCA2 have been implicated in hypergravity responses [34]. Contribution of other, yet uncharacterized channels in gravity sensing is certainly not excluded either. In fact, pharmacological studies of gravitropism have suggested a role for Ca^2+^ transients in gravity signal transduction. Treatments with extracellular Ca^2+^ chelators, Ca^2+^-channel blockers inhibitors of calmodulin and/or Ca^2+^/calmodulin-dependent protein kinases (such as KN-93), all resulted in altered gravitropism [35,36]. Unfortunately, the temporal and spatial resolution of these earlier studies was insufficient to resolve which phase of gravitropism is affected by Ca^2+^. In fact, recent experiments revealed a role for Ca^2+^ in modulating the extracellular pH changes that occur along gravistimulated Arabidopsis root tips in response to auxin, a later phase in the gravitropic response [37].

Inositol 1,4,5-trisphosphate (InsP_3_) has been identified as a potential gravity signal transducer in both shoot and root statocytes, which may be consistent with a role for Ca^2+^ in gravity signaling. InsP_3_ levels were found to first fluctuate, then increase at the bottom half of oat coleoptiles and *Arabidopsis* inflorescence stems upon gravity sensing [38]. Overexpression of human inositol trisphosphatase, which hydrolyzes InsP_3_, in *Arabidopsis* roots, stems, and hypocotyls, caused altered gravitropism [38]. Similarly, inhibiting the synthesis of InsP_3_ with U73122 (a phospholipase C inhibitor) led to altered root gravitropism [39]. On the other hand, mutations affecting the *Arabidopsis INOSITOL POLYPHOSPHATE 5-PHOSPHATASE 13* gene enhanced gravitropism while reducing endomembrane trafficking [40]. Altogether, these data are consistent with a role for InsP_3_ in gravity signaling in both shoots and roots. Whether this regulatory process occurs early in the transduction pathway within the statocytes, or late during auxin-gradient translocation to the elongation zone or curvature-response, remains unclear [38]. Furthermore, the connection between InsP_3_ and Ca^2+^ signaling is less clear in plants than it is in animals [41].

If Ca^2+^-selective, mechanosensitive ion channels truly contribute to gravity sensing in the statocytes, we would expect observing Ca^2+^ transients after gravistimulation. Experiments involving the transgenic AEQUORIN Ca^2+^-reporter system demonstrated the existence of biphasic spikes in cytosolic Ca^2+^ within seconds of gravity stimulation, with only the second peak being sensitive to gravistimulation [42,43]. Analysis of these spikes under short periods of hypo- and hyper-gravity during parabolic flights led to a modified starch-statolith hypothesis suggesting an important contribution of connections between sedimenting amyloplasts and cytoskeletal elements in gravity sensing [33]. While very interesting, these conclusions remain tentative because these Ca^2+^ spikes could not be unambiguously assigned to specific cell types [43]. In addition, they derived mainly from hypocotyls and petioles, and could not be detected in roots.

A different Ca^2+^ detection method was recently used to measure changes in cytosolic calcium levels in the statocytes of *Brassica napus* roots exposed to gravistimulation within the microgravity confines of the International Space Station [44]. Seedlings were grown either under microgravity or exposed to centrifugation simulating Earth’s gravity, then fixed and subsequently used to evaluate both amyloplast sedimentation and cytoplasmic Ca^2+^ levels using a pyroantimonate precipitation method. This experiment demonstrated changes in cytosolic Ca^2+^ accumulation in the statocytes upon both gravistimulation onset and removal, despite only minor changes in amyloplast location [44]. While these results are encouraging, it will be very important to rule out the possibility of experimental artifacts with in vivo methods that allow detection of Ca^2+^ changes in the statocytes of live tissues as they respond to gravistimulation.

One major implication of the amyloplast-pressure model of gravity sensing is gravitropism should be sensitive to the intensity of gravistimulation [45]. However, clever clinostat-centrifugation experiments showed a shoot gravitropic insensitivity to gravity intensity under both hypo- and hyper-gravity conditions for four representative angiosperm species [45]. Instead, the gravitropic responses were only dependent on the angle of inclination from the gravity vector, suggesting inclination rather than gravitational force or acceleration, might be sensed by shoot statocytes (*inclination-sensor model*) [45]. It seems reasonable to speculate similar properties for root gravitropic sensitivity [45,46,47].

Plant organs are capable of responding to very small inclinations from the vertical, a behavior that would not be expected if the sensor were made of granular particles (the amyloplasts) that sediment to the bottom of the statocytes like sand grains [48]. Indeed, such particles should start sedimenting only at threshold inclination angles due to friction and interparticle jamming [48]. Yet, a careful investigation combining a kinematic analysis of statolith sedimentation in plant cells along with studies of heavy Brownian particles movement in suspension within microfluidic cavities serving as cell mimics, demonstrated that amyloplasts can move in response to very small inclination angles, a behavior reminiscent of granular liquids. This surprising behavior may be a consequence of the high levels of statolith agitation/saltation found in live statocytes, which result from cellular activity (see Section 2.1. of this review) [48]. Hence, the characteristics of amyloplast movement in the statocytes are compatible with an inclination-sensor model of gravity sensing.

Considering this conclusion, what are the molecular mechanisms that might contribute to inclination sensing in plants? One possibility is statocytes sense the proximity of amyloplasts with specific cellular structures at the sides of the cells, rather than detecting the pressure exerted by sedimenting amyloplasts on specific membranes. For example, such an inclination-sensor model could involve the interaction between a ligand carried by sedimenting amyloplasts and receptors present in sensitive membranes on the sides of the statocytes. Such a ligand-receptor model was originally proposed to explain the gravitropic response of single-cell rhizoids in the green algae *Chara*. In this system, simple contacts between sedimenting statoliths and sensitive membranes on the sides of the cells appear sufficient for gravity sensing; a force is not needed [46]. A similar model was also proposed to explain gravity sensing by sedimenting amyloplasts in angiosperm statocytes [47]. An alternative mechanism could involve functional interactions between the group of sedimented amyloplasts and the vesicle trafficking machinery that is critical for proper location of auxin transporters [49]. Either way, it is interesting to note the inclination-sensor model allows organ responses to very small inclinations from the vertical while preventing excessive reactions against wind, rain, and other vibrations created by environmental stimuli [49].

The molecular identities of the hypothesized ligand(s) and gravitropic receptors contributing to gravity sensing in plant statocytes remain unknown. However, we do know activation of this pathway upon plant reorientation within the gravity field leads to an asymmetrical redistribution of auxin across the stimulated organ. We will now review the molecular mechanisms that may contribute to this process. First, we will briefly summarize what we know about the molecular mechanisms that contribute to auxin transport in angiosperm roots.

## 3. Gravity Signal Transduction in the Root Statocytes

### 3.1. Auxin Transport in Plant Tissues Involves Auxin Influx and Efflux Carriers

As discussed in the following sections, gravity sensing within the root statocytes triggers the lateral transport of auxin across the root cap, with accumulation at the bottom flank. The resulting lateral auxin gradient is then transported to the EZ where it is responsible for gravitropic curvature. Before we discuss the molecular mechanisms that contribute to polarization of lateral auxin transport within the root cap statocytes in response to gravistimulation, we will introduce the reader to the machinery that mediates auxin transport.

In angiosperms, auxin is mostly synthesized in young shoot tissues. From there, it is transported through the vasculature towards the root tip where it mixes with locally synthesized auxin to form a pool that accumulates in the quiescent-center cells and upper tiers of columella cells. From this auxin-maximum center, auxin is redistributed laterally to more peripheral tissues of the root cap, then transported back toward the root elongation zone where it inhibits cell elongation [9].

Auxin transport occurs through columns of cells. Each cell within a column takes up auxin from the apoplast and exports it in a polar fashion. As a weak acid, the main form of auxin in plants, indole-3-acetic acid (IAA), is somewhat protonated in the acidic environment of the apoplast (pH ~5.6), allowing its free diffusion through the plasma membrane [50]. However, most of the apoplastic auxin molecules remain ionized, preventing their diffusion through membranes. Thus, auxin influx carriers are needed for import into the cells. In Arabidopsis, auxin influx carriers of the AUX1/LAX family mediate auxin import [51].

When reaching the cytoplasm, which is neutral, most auxin molecules become ionized a process that prevents free diffusion through the plasma membrane toward the apoplast. Therefore, auxin efflux carriers including members of the PIN family, as well as P-glycoprotein-type transporters, are necessary for auxin export [52,53]. Interestingly, the PIN proteins are polarly localized within transporting cells, providing direction to the flow of auxin. In Arabidopsis roots, the PIN1, PIN3, PIN4, and PIN7 proteins mediate the transport of auxin through the vasculature and pro-vasculature cell files from the shoot into the root tip. The PIN2 protein then contributes to shootward auxin transport through lateral cap and epidermal cells of the root tip where the protein localizes preferentially on the shoot-facing side of the transporting cells. Additionally, the PIN2 protein is also expressed in the cortical cells of the transition zone and EZ, where it localizes on the shoot facing side at the proximal EZ. Interestingly, PIN2 also localizes on the tip-facing and inner sides at the DEZ, contributing to an auxin reflux from the shootward stream back into the tip-directed stream [54]. An alternative cell-to-cell transport via plasmodesmata also enables auxin reflux to the root tip and its accumulation in cells of the quiescent-center and upper columella layers [55].

**Figure 1:**
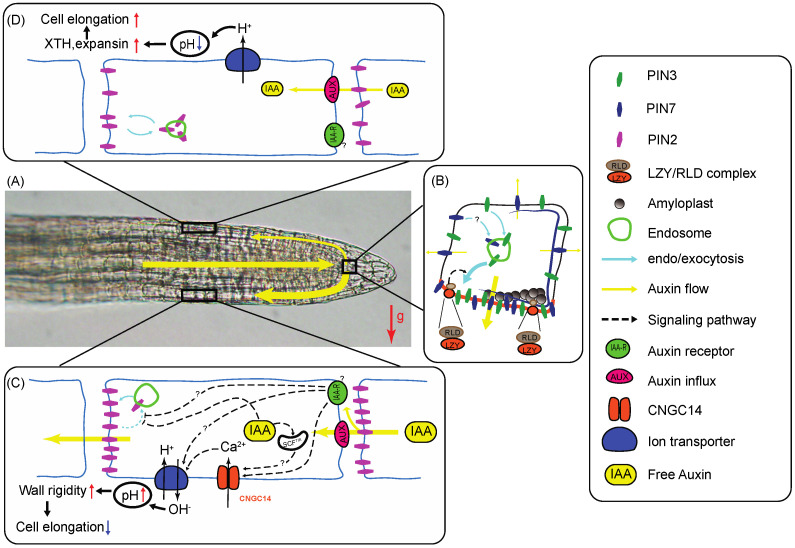
Model illustrating some of the molecular mechanisms that underlie root gravitropism in flowering plants. (**A**) An *Arabidopsis thaliana* root tip is positioned horizontally (gravistimulated). Auxin flow along the root tip is indicated with yellow bars whose widths represent flow intensity. In roots, auxin is transported from the shoot through the vasculature into the root tip where it is redistributed laterally to peripheral tissues and transported back to the elongation zone (EZ). The gravity vector (g) is represented by a red arrow. (**B**) Upon gravistimulation, amyloplasts sediment within the columella cells of the root cap, triggering a signal transduction pathway that leads to LZY protein association with the plasma membrane where it recruits RLD regulators of vesicular trafficking. RLD then directs PIN3 (and possibly PIN7) transcytosis toward the lower membrane, resulting in preferential downward transport of auxin and lateral auxin gradient formation across the cap [68]. (**C**,**D**) The auxin gradient generated across the cap upon gravistimulation is transported to the elongation zone where it promotes differential cell elongation between opposite sides and downward curvature. (**C**) Epidermal cells on the lower side of the root elongation zone (EZ) are exposed to high auxin levels, which promote PIN2 retention at the plasma membrane through a pathway that involves uncharacterized receptors. Increased auxin levels also activate CNGC14 ion channels, leading to increased cytosolic Ca^2+^ levels and activation of a H^+^/OH^-^ antiporter. This pathway leads to an increase in apoplastic pH responsible for wall-polymer crosslinking and inhibition of cell elongation [69]. The plasma membrane associated auxin receptors (labeled IAA-R) involved in these processes remain poorly characterized. (**D**) In the upper half of the root, transporting cells are exposed to lower auxin levels, resulting in a decreased retention of PIN2 within the plasma membrane and lower auxin transport potential on this side of the root. Furthermore, decreased auxin levels lead to the activation of H^+^ pumps, resulting in an acidification of the apoplast. Decreased apoplastic pH leads to the activation of expansin and XTH enzymes responsible for increased elongation. The combination of decreased expansion on the lower side and increased elongation on the upper half results in downward root curvature at the distal side of the elongation zone. The molecular players are represented by symbols whose identity is revealed in the legend on the right of the figure. Yellow arrows indicate auxin transport. Dotted arrows represent signal transduction pathways. Blue arrows represent endo- and exocytosis. Question marks identify molecules or steps within pathways that are not well characterized. The various steps represented in this model are described and discussed in the text with appropriate references. Contribution of canonical nuclear SCF^TIR^-dependent expression regulation to the pathways described here is not represented in this figure. This figure was inspired from [68,69,70].

Key to gravitropism is a statocyte-localized lateral auxin redistribution system that connects the auxin-maximum center at the middle of the root cap to the peripheral shootward auxin stream that takes place in the lateral cap and epidermal cells of the root tip. The *PIN3* and *PIN7* genes are expressed in overlapping domains of the root cap columella region (upper two tiers of columella cells for *AtPIN3*, and tiers 2 and 3 for *AtPIN7*; Figure 1A,B), where they contribute to lateral auxin transport.

In vertical roots, the PIN3 and PIN7 proteins are distributed uniformly to the plasma membrane on all sides of the cells, allowing for symmetrical auxin redistribution to the lateral cap. However, upon plant reorientation within the gravity field, these proteins quickly relocalize to the lower side of the statocytes, thereby driving auxin toward the lower flank of the root cap [56,57]. Gravistimulus-induced relocalization of the PIN3 and PIN7 proteins to the bottom side of the statocytes occurs through a transcytotic process dependent upon small GTPases of the ADP-ribosylation factor (ARF) type, associated with GNOM-type GDP/GTP exchange factors (GEFs) [57,58].

Gravity-induced PIN protein polarization and auxin transport activity within the statocytes appear to be modulated by PIN-protein phosphorylation via plasma membrane-associated AGCVIII-type Ser/thr protein kinases at conserved residues. Amongst these kinases, the PINOID(PID)/WAVING AGRAVITROPIC ROOTS (WAG) kinases modulate both polar localization and auxin transport [59,60,61,62]: Indeed, PID over-expression led to decreased PIN3 polarization upon gravistimulation in both root and hypocotyl statocytes along with altered gravitropism, whereas knockout mutations led to increased PIN3 relocalization in the columella cells upon gravistimulation [63,64]. Hence, the PID/WAG kinases function as negative regulators of gravity-induced PIN3 relocalization in the statocytes. D6 PROTEIN KINASES (D6PKs) also phosphorylate PIN proteins at a set of conserved sites overlapping with PID/WAG targets [60,62]. Interestingly, knocking out D6PKs in Arabidopsis resulted in hypocotyl gravitropism defects associated with decreased PIN-protein phosphorylation. Phototropically-induced PIN3 relocalization was not affected, suggesting D6PKs modulate only auxin transport-activity [62].

Taken together, these results suggest phosphorylation events modulate both gravity-induced PIN relocalization in the statocytes and/or their auxin-transport activity. It has been suggested gravity-induced PIN3/7 relocalization within the statocytes drives the formation of a lateral auxin gradient, ultimately leading to the gravitropic curvature. However, we cannot yet exclude the possibility gravity-induced PIN3/7 relocalization to the bottom side of statocytes is a consequence of differential activation of auxin efflux [65]. Such a functional activation could be triggered by differential phosphorylation on the bottom side of the cells, or another regulatory mechanism that remains uncharacterized.

It is interesting to note higher auxin levels in the apoplast have been associated with increased retention of auxin-efflux transporters within the plasma membrane [66]. Furthermore, the statocytes have been shown to display rapid increases in cytoplasmic pH accompanied by apoplast acidification early after gravistimulation [67]. This acidification of the apoplast is critical for full gravi-responsiveness, possibly because it is responsible for a decrease in the fraction of auxin taking an ionized form in the apoplast, thereby facilitating its mobility through membranes [67].

While important for gravitropism, the process of gravity-induced PIN3/7 relocalization within the statocytes is neither essential nor sufficient for the response. Indeed, not all roots stimulated by gravity will display evidence of PIN3/7 relocalization in the statocytes [65]. Furthermore, not all statocytes within a stimulated root show evidence of PIN3/7 polarization [65]. Also, *pin3* and *pin7* single mutants show only slight defects in gravitropism, whereas the double mutants display stronger but still only partially agravitropic phenotypes. Together, these results suggest other transporters may be important for gravity-induced auxin-gradient formation across the root cap.

### 3.2. Gravity-Signal Transducers Contribute to Vesicular Trafficking and Plastid Function

Genetic approaches have been effective at identifying some of the molecular mechanisms that contribute to gravity sensing and initial phases of gravity signal transduction in the root statocytes. The first mutations shown to affect gravity signal transduction in *Arabidopsis thaliana* were named *altered response to gravity1* (*arg1*) and *arg1-like2* (*arl2*). Mutant seedlings displayed strong, though partial, defects in root and hypocotyl/shoot gravitropism accompanied by alterations in gravity-induced cytoplasmic alkalinization of the statocytes and an absence of PIN3 relocalization upon gravistimulation [65,71,72,73]. They displayed wild-type root-growth responses to auxin and auxin-transport inhibitors and their statocyte amyloplasts sedimented like wild type [65,74]. Taken together, these results are compatible with a role of *ARG1* and *ARL2* in gravity signal transduction within the statocytes, a conclusion reinforced by observations of full phenotypic rescue by targeted expression of a wild type transgene specifically in the statocytes of mutant seedlings [74]. The ARG1 and ARL2 genes were shown to encode paralogous J-domain-containing proteins that associate with the plasma membrane and multiple compartments of the vesicle trafficking pathway, modulating PIN protein relocalization within the statocytes [65,74].

More complex genetic screens taking advantage of the partially agravitropic phenotype displayed by *arg1* mutant seedlings resulted in the identification of two genetic enhancers of *arg1* named *modifier of arg1-1* (*mar1-1*) and *mar2-1*. *mar1-1* is a hypomorphic allele of *TOC75*, a gene that encodes the channel component of plastidic TRANSLOCON ON THE OUTER CHLOROPLAST MEMBRANE (TOC) complex, which mediates the import of cytoplasmic proteins through the outer membrane of plastids. *mar2-1* is an hypomorphic mutation of *TOC132*, which encodes another component of the same TOC complex [75]. Interestingly, these *mar* mutations did not obliterate protein import into the plastids. In fact, mutant root-cap amyloplasts accumulate starch and sediment at wild-type rates upon gravistimulation. This suggests amyloplasts may be playing a more active role in gravity signal transduction, beyond simply sedimenting like rocks in the statocytes [47,75].

A ligand-receptor model of gravity sensing postulating sedimenting amyloplasts might carry ligands that interact with receptors on the side of the statocytes to trigger gravity signal transduction, was proposed to explain these observations. However, TOC132 was subsequently ruled out as a possible ligand based on a structure-function analysis of its role in gravitropism [47]. A differential proteomic analysis comparing wild-type and *toc132* mutant roots identified candidate gravity signal transducers, whose functions remain to be characterized [47].

Another proteomic analysis of gravity-responding root-tips identified ADENOSINE KINASE 1 (ADK1) as a possible gravity signal transducer. This enzyme catalyzes the phosphorylation of adenosine, a metabolite that is produced by the methyl-donor (AdoMet) cycle and nucleic acid metabolism. A mutation in the *ADK1* gene strongly affects root gravitropism and PIN3 protein localization in the root cap without altering gravity-induced cytoplasmic alkalinization. Therefore, *ADK1* may regulate a step of gravity-signal transduction that controls PIN-protein relocalization within the statocytes but does not contribute to their cytosolic pH responses [76]. Unfortunately, its mode of action remains largely uncharacterized.

Probably the most exciting recent development in our investigations of gravity signal transduction in plants is the identification of the *LAZY* genes and the initial molecular characterization of their roles in gravity signal transduction. The *lazy* mutation was originally identified as resulting in a prostrate shoot phenotype in rice without affecting amyloplast sedimentation in the statocytes [77,78]. *lazy* mutant coleoptiles failed to develop a lateral auxin gradient upon gravistimulation [79]. Subsequent molecular characterization revealed a *LAZY1* gene that encodes a plant-specific protein of unknown function (see below). *LAZY1* orthologs have been identified and initially characterized in several plant species. In the legume model *Medicago truncatula,* mutations named *negative gravitropic response of roots* (*ngr*), which lead to upward growing roots, were found to affect a *LAZY1* ortholog [80].

Six *LAZY*-like genes have been identified in *Arabidopsis thaliana* [80]. Four of the six *Arabidopsis LZY* genes (*LZY1-4*) were found to be expressed in the statocytes whereas *LZY5* and *LZY6* transcripts could not be found [81,82,83]. Arabidopsis *LZY2* and *LZY3* are expressed in the statocytes of both shoots and roots whereas *LZY1* is mostly expressed in the shoot statocytes (endodermis) and *LZY4* in the root tip [81,82,83,84]. Phenotypic analysis of single, double and triple mutants in these genes provided direct evidence of functional redundancy [80,81,82]. For instance, single mutations in *LZY3* resulted in altered lateral root growth angle, a phenotype that was exacerbated by bringing the *lzy2* mutation in the same background. This phenotype was further enhanced to an upward root phenotype when the *lzy4* mutation was added [80,81]. Significantly, targeted expression of *LZY1* within the statocytes of *lzy1 lzy2 lz3* triple mutant plants, which display agravitropic primary stems and downward lateral branches, allowed full rescue of the gravitropic defect, further confirming functional redundantly between members of *LZY* gene family in Arabidopsis statocytes [82].

The gravitropic defects displayed by *lzy1 lzy2 lzy3* mutant shoots and *lzy2 lzy3 lzy4* mutant roots were associated with impaired lateral auxin transport, whereas amyloplast sedimentation in the statocytes remained unaffected [80,81,82]. Furthermore, Arabidopsis *lzy1 lzy2 lzy3 lzy4* quadruple mutants were shown to display normal phototropism, a response that is also dependent upon PIN-mediated lateral auxin transport [81]. Together, these results confirmed a role for LZY proteins in gravity signal transduction within the statocytes, leading to lateral auxin transport following amyloplast sedimentation. So, how do the LZY proteins contribute to this process?

There is currently no clear answer to this question. However, investigations of protein localization and sequence, and identification of interacting partners, may provide meaningful clues to this process. Expression of LAZY proteins fused with GFP showed the proteins localize at the cell periphery, in association with plasma membrane markers, and within the nucleus [85,86,87,88,89]. Furthermore, fusions of Arabidopsis LZY3 with mCherry gave detectable fluorescence signals in the lateral root cap columella cells upon fixation and clearing while being invisible in the primary roots. In lateral root columella cells, the LZY3-mCherry signal was associated with the plasma membrane on the lower side of the cells in the absence of a gravistimulus. Upon plant reorientation within the gravity field, the mCherry signal relocalized to the new bottom side of the cell, still within the plasma membrane, within 30 min of stimulation, a time that was also sufficient for amyloplast sedimentation to occur. Hence, LZY3 protein relocalized to the lower membrane of lateral root statocytes in response to gravistimulation [68].

Protein sequence comparisons between *LAZY* orthologs found in multiple plant species revealed the existence of 5 conserved domains within the protein, named I through V (from amino- to carboxy-termini, respectively). The functional significance of these regions for plant gravitropism has been investigated by mutating each region within the Arabidopsis *AtLAZY1* gene and testing the ability of each mutant allele to rescue the large inflorescence branch angle of mutant plants as well as localizing the mutant protein in plant cells [90]. Results demonstrated region I is needed for AtLAZY1 protein association with the plasma membrane and its function in gravitropism. Mutating regions III and IV had little impact on the protein function in gravitropism and its localization within the plasma membrane. Interestingly, altering region II with changes in two conserved amino acids resulted in a switch of shoot gravitropism from negative to positive, a phenotype associated with reversed auxin gradient formation across lateral stems upon gravistimulation. Hence, region II appears important for the control of shoot GSA, although its mode of action remains uncharacterized. Mutations in region V disrupted AtLAZY1 function in shoot gravitropism without affecting its association with the plasma membrane of statocytes [90].

Recent functional analysis of region V, also named CCL motif, revealed a possible role for LZY3 protein in recruiting regulators of polar auxin transport to the plasma membrane at the lower side of lateral root cap statocytes upon gravistimulation [68]. In Arabidopsis, expression of carboxy-deleted versions of the *LZY2* and *LZY3* lacking the CCL domain failed to rescue the gravitropism defect of *lzy1 lzy2 lzy3* mutant seedlings [82]. Furthermore, over-expression of the CCL domain in the statocytes of *lzy1 lzy2 lzy3* mutant plants resulted in upward-growing roots, a phenotype similar to that of *lzy2 lzy3 lzy4* mutant seedlings [82]. These exciting results were interpreted to suggest over-expressed CCL peptide may compete with full-length LZY4 protein for binding to a factor that is critical for root gravitropism [82].

LZY CCL-domain-interacting partners were recently identified as RCC1-LIKE DOMAIN (RLDs) proteins, which are conserved amongst land plants and share several domains including a *Pleckstrin Homology* (PH) domain, *Regulator of Chromosome Condensation 1* (RCC1)-Like repeats, a *Fab1/YGL023/Vps27/EEA1* (FYVE) domain, and a *Brevis Radix* (BRX) domain. The latter BRX domain of RLD proteins was found to directly interact with the CCL domains of Arabidopsis LZY2 and LZY3 proteins. In Arabidopsis, four *RLD* genes (*RLD1-4*) are expressed in the root cap and vascular tissues of the main and young lateral roots. *rld1 rld4* double mutants displayed decreased primary root gravitropism and increased lateral root GSA, phenotypes that are associated with decreased PIN3-GFP signal in lateral root-cap statocytes. Furthermore, *rld1 rld2 rld3 rld4* quadruple mutants displayed severe embryo-development defects associated with altered vasculature development and decreased PIN1-GFP signal. Together, these data suggest RLDs regulate auxin flow through PIN proteins in both root gravitropism and plant development.

Careful kinetic investigations of cellular localization of functional fluorescent protein tagged versions of LZY3, RLDs and PIN3 within the lateral root cap statocytes of control and gravistimulated wild type and mutant seedlings strongly suggested the LZY3 protein relocalizes to the bottom side of the lateral root cap statocytes upon gravistimulation, where it recruits RLDs. This process then triggers a repolarization of the PIN3 protein to the same side of the statocytes, leading to lateral auxin transport [68] (Figure 1B).

The RLD proteins share a 300–500 amino acid domain (the RLD domain) capable of catalyzing guanine nucleotide exchange on Rab8a, a protein that contributes to post-Golgi trafficking to the plasma membrane along with the exocyst complex [68,91,92]. Therefore, it is reasonable to speculate RLD recruitment by LZY3 to the plasma membrane on the lower side of gravistimulated lateral root statocytes may regulate the trafficking of PIN3 auxin efflux facilitators to that side of the statocytes, leading to lateral auxin transport, auxin gradient formation, and gravitropic curvature [68].

The mechanisms contributing to gravity signal transduction in lateral roots differ significantly from those implicated in primary root gravitropism, as discussed in Section 6 below. However, it should be pointed out the phenotypic analysis of single, double, and multiple *lzy* mutants demonstrated a role for *LZY3* in primary root gravitropism as well, redundant with other members of the *LZY* gene family (see above). Furthermore, *rld1 rld4* double mutants displayed decreased primary root gravitropism in addition to their lateral root phenotype [68]. Although the LZY3-mCherry protein was only detected in lateral root statocytes, absence of a fluorescence signal in the primary root tips is not evidence of LZY3 absence from these tissues as LZY proteins are notoriously difficult to detect in plant cells [90]. From these observations, we suggest this process may also be functioning in primary root statocytes, although more work will be needed to test this conclusion [68].

One key question that remains unanswered, however, relates to the molecular mechanisms that govern LZY3 protein binding to the plasma membrane in the statocytes and its polarization to the lower side upon gravistimulation. Whether this association is mediated by protein-protein or protein-lipid interactions remains unknown, although the authors speculate the K/R-rich domain found within LZY proteins could contribute to interactions with positively charged anionic phospholipids [68]. It is worthwhile recalling here that multiple studies have suggested a role for the phosphoinositide pathway in root and shoot gravitropism (see Section 2 and Section 3 above). Whether this involvement of the phosphoinositide pathway in gravitropism has anything to do with LZY protein relocalization to the lower plasma membrane of the statocytes remains unknow.

Observations of interactions between LAZY and BRX domain-containing proteins are not limited to *Arabidopsis thaliana*. Indeed, rice OsLAZY1 was also found to interact with OsBRXL4 at the plasma membrane [88]. This interaction is relevant to gravitropism because silencing of *OsBRXL* genes by RNA interference led to a compact plant phenotype due to narrower tiller angles whereas OsBRXL4 over-expression resulted in a prostate growth phenotype due to wider tiller angles [88]. While LAZY-BRXL interactions occur at the plasma membrane and are needed for polar auxin transport, the rice system appears to differ from the Arabidopsis system in the sense that nuclear localization of rice LAZY1 is necessary for its function in gravitropism [88]. It is worthwhile noting that the sequence of LAZY domain V also matches that of an *Ethylene-Responsive Element Binding Factor-Associated Amphiphilic Repression* (EAR) motif, which has been associated with transcriptional repression [87,89]. In fact, the wheat TaDRO1 LAZY-like protein was found to interact with wheat TOPLESS, a protein whose Arabidopsis ortholog contributes to transcriptional repression of auxin-responsive genes by binding to AUX-IAA proteins [89]. In the future, it will be important to determine if these associations are relevant to gravitropism in monocot plants. It is unlikely they do so in Arabidopsis because mutations in the nuclear localization signal of AtLZY1 do not affect its ability to function in gravitropism [84].

It is well established multiple environmental cues such as light and obstacles modulate the gravitropic response (reviewed in [70]. An interesting pathway modulating the interaction between light responses and root gravitropism was recently described in *Arabidopsis thaliana*, involving a LAZY protein. In roots, light was shown to promote expression of *HY5*, which binds to enhancers within the *LAZY4* gene promoter, leading to increased *LAZY4* expression and enhanced positive root gravitropism. In darkness, low *HY5* expression resulted in decreased starch accumulation in the statocytes, decreased *LAZY4* expression and reduced root gravitropism [93]. Interestingly light-dependent regulation of expression of another class of transcription factors, the PHYTOCHROME INTERACTING FACTORS (PIFs), was shown to have opposite effects in hypocotyls, with exposure to light resulting in PIF degradation, decreased *LAZY4* expression and inhibition of negative hypocotyl gravitropism [93]. Hence, light-dependent regulation of expression of key transcription factor genes allows modulation of the relative roles of gravitropism and phototropism in directing the growth of hypocotyls and roots.

In conclusion, genetic investigations of root gravitropism have identified a number of loci that contribute to the transduction of information provided by amyloplast settling into a transcytotic process that targets PIN proteins to the bottom side of the statocytes. LAZY proteins associate with the lower membrane, recruiting RLD regulators of vesicular trafficking that ultimately target the PIN3 auxin efflux facilitator to the lower membrane. This leads to the formation of a lateral auxin gradient across the cap that ultimately promotes a curvature response upon transmission to the EZ.

## 4. Auxin Gradient Propagation from Root Cap to EZ

The mechanisms discussed above allow the formation of a lateral auxin gradient across the root cap upon gravistimulation. However, the curvature response that ensues involves the differential elongation of cells between upper and lower root flanks at the distal side of the elongation zone (DEZ). This implies the gravistimulus-induced lateral auxin gradient has to be transmitted from the root cap to the EZ for this curvature to occur. The mechanisms contributing to auxin gradient translocation toward the DEZ have been well investigated, leading to the conclusion this gravity-induced auxin gradient is not only propagated quite effectively between site of gradient formation and locale of curvature response, but it is also expanded in the process.

In roots, the shootward transport of auxin occurs in peripheral tissues including the lateral root cap, epidermis and cortex (Figure 1C,D). In these tissues, transporting cells take up auxin mostly through AUX1/LAX-type influx carriers as well as free diffusion through the plasma membrane, and export it through polarly localized PIN2 efflux carriers as well as members of the p-glycoprotein family (see Section 3.1). The shootward localization of PIN2 within the lateral cap and epidermal cells dictates the shootward movement of auxin within those tissues, whereas its lateral and rootward localization in cortical cells at the distal side of the EZ allows an auxin reflux towards the vasculature and root cap [94]. Both flows are critical for an efficient gravitropic response [94]. Reversible phosphorylation modulates PIN2 localization within transporting cells, with the serine/threonine protein kinases PID, WAG1, and WAG2 contributing to its phosphorylation, and type-IIA protein phosphatase complexes (PP2A) contributing to its dephosphorylation. Phosphorylated PIN2 localizes at the rootward side of the transporting cells, whereas dephosphorylated PIN2 associates with the shootward side of the cell [95,96].

Like other PIN proteins, PIN2 is constantly recycled between plasma membrane and endosome (Figure 1C,D). The amount of PIN2 protein retained at the plasma membrane within epidermal and lateral root cap cells increases with auxin availability [66]. Consequently, auxin accumulation on the bottom side of a gravistimulated root tip leads to a transient increase in auxin transport potential on that side, and lower transport potential on the upper side, thereby increasing steepness of the lateral auxin gradient that was generated across the root cap upon gravistimulation as it progresses toward the EZ [66,97,98]. This process is exacerbated by increased auxin-dependent production of small signaling peptides *GOLVEN1* (*GLV1)* and *GLV2* by cells on the lower side, which trigger a response pathway that also favors PIN2 association with the plasma membrane [99]. Furthermore, other phytohormones, such as the brassinosteroids and gibberellic acid, also function as antagonists of PIN endocytosis [100]. On the other hand, a long period of exposure to higher auxin levels triggers vacuolar sorting of the PIN2 protein and its vacuolar degradation, thereby potentially contributing to gradient dissipation at the end of a gravitropic response [66]. Hence, a complex crosstalk between hormonal signaling and PIN2 endocytic sorting contributes to regulating root gravitropic curvature [100].

The auxin-induced increase in alkalinization of the apoplast on the lower side of a graviresponding root may also contribute to auxin gradient regulation. Indeed, it may trigger a decrease in the fraction of protonated IAA molecules in the apoplast, thereby decreasing the rate of free diffusion through the plasma membrane of transporting cells. Consequently, the auxin influx carrier AUX1 is needed for adequate shootward auxin transport and root gravitropism [37,101]. The *AUX1* gene is broadly expressed in the root tip, including the provasculature, root-cap, and epidermal cells. However, restricting its expression to the lateral-cap and epidermal cells of the root tip is sufficient to rescue the altered root gravitropism phenotype displayed by *aux1* mutant seedlings, indicating it functions in these tissues to modulate root gravitropism [101,102].

Considering the preceding discussion, which strongly emphasizes a key role for Arabidopsis PIN2 in directing rootward transport of auxin from cap to elongation zone during gravitropism, it is quite surprising *Arabidopsis pin2* mutant roots retain some gravitropic capability [9]. This suggests other auxin transporters may function redundantly with PIN2 to direct auxin transport in peripheral tissues of the root tip. In fact, P-glycoprotein-type transporters, which use ATP hydrolysis to carry target molecules through membranes, may fulfill some of this redundant auxin-transport function. Indeed, *Arabidopsis AtPGP1* and *AtPGP19* are also expressed in the root EZ, and functional studies in heterologous systems such as plant protoplasts, yeast and mammalian cells, have demonstrated their ability to transport auxin [53,103,104].

## 5. The Gravitropic Curvature Results from Differential Cell Elongation between Opposite Flanks of the Root EZ

The auxin-transport mechanisms described in the previous section allow transmission of the gravistimulus-induced lateral auxin gradient between the root cap and the EZ where it triggers differential cellular elongation between upper and lower flanks within 10 to 15 min of stimulation, leading to downward gravitropic curvature. The epidermis appears to be the main driver of root-tip curvature. Indeed, expressing an auxin-response repressor (*axr3-1)* in epidermal cells of the EZ is sufficient to obliterate the gravitropic response whereas expressing it in different cell types has little impact [102]. Therefore, we now must discuss the molecular mechanisms that allow epidermal cells at the EZ to respond to a lateral gradient of auxin by differentially elongating between upper and lower sides upon gravistimulation.

The effects of auxin changes on cell elongation along the root tip have been investigated. Increased auxin levels such as those identified at the lower flank of gravistimulated roots appear to activate the CYCLIC NUCLEOTIDE GATED Ca^2+^ 14 (CNGC14) channel, leading to Ca^2+^ influx into the cytoplasm and consequent activation of a plasma-membrane H^+^/OH^−^ conductance (Figure 1C). These rapid conductance changes result in an alkalinization of the apoplast accompanied by an increase in cell-wall rigidity and decreased rate of cellular elongation [37,105]. This fast response was suggested to bypass a need for auxin influx into the cytoplasm [69]. However, a similar response in root hairs was more recently shown to involve auxin uptake through AUX1/LAX transporters followed by activation of a SCF^TIR1/AFB^ receptor complex within the cytoplasm [106]. Whether such a cytoplasmic receptor complex is also involved in auxin sensing within the EZ epidermal cells remains to be determined.

Other signaling molecules also contribute to the regulation of EZ cell elongation in response to auxin. For instance, nitric oxide (NO) accumulates on the lower side of gravistimulated roots where it inhibits auxin transport and modulates auxin signaling through S-nitrosylation of TIR1 [107]. Similarly, reactive oxygen species (ROS) have been shown to increase at the bottom side of roots in an auxin-dependent manner, where they contribute to the regulation of gravicurvature [108].

The upper flank of the EZ is exposed to lower auxin levels upon gravistimulation, resulting in increased cell-wall acidity (Figure 1D). Increased acidity in the apoplast leads to the breakage of intermolecular cross-links between wall polymers by expansins and xyloglucan endotransglucosylases/hydrolases (XTHs), favoring cellular elongation [37].

Ultimately, increased cell elongation on the topside and decreased elongation at the bottom result in downward tip curvature [37,109]. This curvature will have to proceed until the tip has returned to the vertical. Yet, experiments involving the use of in situ auxin sensors, such as *DR5-GFP* and *dII-VENUS,* have demonstrated the auxin gradient generated across the root cap and transmitted to the EZ disappears when the tip reaches an approximate angle of 50^0^ from vertical. At this angle, the amyloplast statoliths have returned to the distal side of the statocytes and the gravity-induced polarity of PIN3/7 proteins has disappeared, being replaced by a symmetrical distribution on all sides of the cells [13]. From this point, the curvature will have to proceed in the absence of a lateral auxin gradient until the tip reaches the vertical. Unfortunately, the molecular mechanisms that contribute to this second auxin-gradient-independent phase of gravicurvature and its termination when the tip reaches the vertical, remain unexplained [13]. One possibility could be this phase of the response involves the secondary, cap-independent mechanism of gravity sensing mediated by the DEZ, as discussed in Section 2 of this review. More work is needed to test this possibility.

## 6. The GSA of Lateral Roots Differs from That of Primary Roots

The architecture of a root system defines its ability to either survey shallow areas of the soil for nutrients uptake or dig deeper underground to seek out buried resources such as water during drought periods. While the primary roots of most plants have a tendency to grow orthogravitropically (parallel to gravity), the lateral roots emerging from the primaries tend to grow either diagravitropically (horizontally), or plagiogravitropically (obliquely), allowing for broader soil exploration for water and nutrients [110].

In *Arabidopsis*, lateral roots emerge at a 90-degree angle from the primary root, then acquire plagiogravitropism as starch builds up within statocyte amyloplasts and the EZ becomes established [111,112,113]. After reaching their initial shallow GSA, the laterals straighten up and continue growing along this GSA for some time. Eventually, these laterals curve again, leaning toward positive orthogravitropism. The length of the period during which laterals follow a specific GSA, and the value of this GSA, define the radial expansion of the root system, a key parameter in establishing plant productivity and survival under specific conditions.

These successive phases of lateral root gravitropism have been investigated in *Arabidopsis thaliana*. Regulation of auxin transport and levels in lateral root statocytes seems responsible for GSA specification. Early after emergence, only *PIN3* is expressed in the columella cells of lateral root caps where it is quickly redistributed toward the bottom side. As discussed in the previous section, PIN3 relocalization is preceded by a polarization of LZY3 to the lower membrane where it recruits RLD proteins from the cytoplasm. RLD controls the vesicular trafficking of PIN3 leading to its polarization to the bottom membrane of the statocytes [68], lateral auxin transport and downward curvature response.

When the young lateral root reaches its first GSA plateau, *PIN3* expression decreases, and *PIN4* and *PIN7* are activated to very low expression levels. At this stage, the overall level of PIN expression in the statocytes is low, and the PIN proteins are symmetrically distributed on all sides allowing the laterals to continue growing straight along the GSA.

This initial phase of plagiogravitropic growth may result from an auxin-dependent antigravitropic offset mechanism that opposes gravitropism to regulate the distribution of auxin levels and response between opposing sides [114]. This offset mechanism is best observed in *lzy2 lzy3 lzy4* triple mutant roots, which show opposite GSA relative to wild type [82,90,115], and in *lzy* mutant plants that express a mutated form of AtLAZY1 carrying two conserved amino acid substitutions in region II of the protein [90]. Its function requires the presence of functional gravity-sensing statocytes with starch-filled sedimenting amyloplasts [116], and involves cytokinin signaling, which interferes with growth at the upper flank of the lateral roots [117].

In addition to the *LAZY1* family of genes, plant genomes also contain other genes that encode proteins sharing region II with LAZY1, but lacking domain V [118,119]. Amongst these additional genes, *TILLER ANGLE CONTROL 1* (TAC1), which encodes a LAZY-like protein lacking region V, was recently found to oppose LAZY by increasing tiller angle in rice, and branch angles in peach trees and Arabidopsis inflorescences in response to light [118,119,120]. TAC1 was proposed to function as an integrator between light perception and LAZY1-mediated gravity response to optimize shoot position for light capture. Unfortunately, a role for TAC1 in regulating the antigravitropic offset mechanism in lateral roots is unlikely because its expression is very low in these organs [116]. Recent identification of three genetic suppressors of *lzy2 lzy3 lzy4* and their characterization may yield interesting new insights into the machinery that governs the antigravitropic offset mechanism in lateral roots [116].

At the end of the plagiogravitropism plateau phase, an increase in auxin levels and signaling within the lateral-root tip leads to a derepression of the *PIN4* and *PIN7* genes, whose proteins polarize to the bottom side of the statocytes. This PIN-protein relocalization leads to the formation of a lateral auxin gradient that is transmitted to the EZ where it is responsible for a new phase of downward curvature [114,121]. Initiation of this final phase of lateral root gravicurvature effectively terminates radial expansion of the root system in *Arabidopsis thaliana*.

In conclusion, several members of the *PIN* gene family modulate distinct phases of radial root-system expansion, a process that may be targeted by developmental and environmental signals that dictate whether a root system will be radially expanded or axially organized [113]. A better understanding of these regulatory processes will be important because they could constitute excellent targets to engineer plants more capable of sustained growth under harsher conditions dictated by climate change or enable cultivation on marginal lands to meet increasing demands from a growing population.

## 7. Conclusions

Recent advances in our understanding of the molecular mechanisms that modulate the various phases of root gravitropism have resulted from careful genetic and cell biological investigations of the process in multiple model organisms. Careful investigations of gravitropic sensitivity to pressure and inclination angles have suggested a mechanism of gravity sensing depends upon inclination rather than pressure, and further work will be needed to identify the molecular players in this process. Several studies have revealed a key role for LAZY-family proteins in linking amyloplast sedimentation to gravity-induced polarization of PIN proteins to the lower membrane of the statocytes. Through recruitment of RLD regulators of vesicular trafficking, these LAZY proteins appear to directly target PIN3 (and possibly also PIN7) transcytosis to the lower membrane, thereby establishing a lateral auxin transport across the root cap ultimately responsible for tip curvature upon transmission to the EZ. It will be very interesting to identify the molecular mechanisms that lead to LAZY targeting to the lower membrane of the statocytes upon gravistimulation. Is it modulated by protein-protein interactions, alterations in lipid composition of the membrane at that location, or other processes such as posttranslational modifications?

Novel discoveries have also expanded our understanding of the molecular mechanisms that allow propagation of the gravity-induced lateral auxin gradient between root cap and EZ, and its enhancement through an auxin-dependent feedback regulation of PIN association with the plasma membrane on the lower side of the root. Here, a better understanding of the auxin receptor(s) responsible for this process is needed. Finally, molecules that transduce the signal associated with the lateral auxin gradient into differential cellular elongation on opposite flanks of the DEZ have been identified and initially characterized. However, more investigations will also be needed to identify the molecules that serve as auxin receptors for this process, their location on the surface and/or within the cell, and their mode of action.

Our understanding of the mechanisms that modulate root gravitropism in response to other environmental cues has also expanded quite substantially in the last few years. In this review, we discussed the involvement of key light-responsive transcription factors related to PIF and HY5 in the regulation of Arabidopsis hypocotyl and root gravitropism via their impact on LAZY4 expression in the corresponding statocytes, thereby enabling phototropism when needed [93]. Effects of drought, osmotic/salt shocks, and darkness on starch accumulation in the amyloplasts of statocytes and gravitropic sensitivity were previously reported [122,123,124]. Furthermore, effects of obstacle detection on PIN-mediated auxin transport to the central region of the EZ (CEZ) to promote a thigmotropic curvature at the CEZ has also been documented [125]. These reports provide interesting insights into some of the mechanisms that allow roots to alter their gravitropic response when they need to favor other tropic behaviors to cope with environmental changes. Exciting new discoveries are likely to follow up on these initial reports, bringing new insights into the molecular mechanisms that allow environmental cues to regulate root gravitropism and/or lateral root GSA, thereby affecting the radial expansion of the root system and its depth. Considering the key role played by root system architecture in modulating plant growth, development, productivity, and environmental adaptation, these research advancements are likely to have profound implications in the near future for our ability to engineer cultivars better suited to cope with the harsher environmental conditions predicted due to climate change, and allow future agriculture expansion into marginal lands to meet an increased demand in food, feed and fiber by rapidly expanding human populations.

Finally, at a time of great hope for space exploration and colonization of other planets, attention is currently given to investigations of plant responses to microgravity, fractional gravity, and hypergravity conditions. This is because plants have been recognized as key components of bioregenerative life-support systems allowing food, feed, and fiber production along with wastewater and air recycling. Better understanding plant responses to altered gravity conditions will allow the development of efficient plant-based bioregenerative life-support systems. Interestingly, experiments under microgravity have revealed new tropic responses to alternative directional cues such as oxygen [126] and light [29,127]. Such tropic responses were indeed predominant in the absence of significant gravity exposure. Furthermore, centrifugation experiments carried out under microgravity conditions to generate fractional gravity, including those present on the Moon or Mars, coupled with transcriptional profiling, have revealed the ability of plant organs to respond to much lower gravitational forces than originally anticipated, possibly as a consequence of graviresistance in addition to the mechanisms described in this review [29].

Exposure to low gravity level, such as that encountered on the Moon (0.18 g) or under microgravity, resulted in developmental abnormalities associated with structural stress at the plasma membrane and cell wall, along with oxidative stress [29,128,129,130,131]. Such stress responses decreased with increased gravity levels, to be almost inexistent under Mars gravity conditions (0.36 g) [29]. Blue light phototropism allowed reduction of the gravitational stress response caused by microgravity [29]. On the other hand, exposure to hypergravity conditions has enabled studies of graviresistance, a process that results in shorter, thicker organs relative to 1g controls (see Section 2.1 above; [28]). While beyond the scope of this review, the results of all these experiments under altered gravity conditions suggest new methods to optimize plant cultivation under distinct g conditions. Therefore, the next few years are likely to dramatically increase our understanding of plant responses to fractional and hypergravity conditions, allowing the development of plant-based bioregenerative life-support systems that will enable exploration of the solar system and colonization of other planets. These are exciting times, indeed!
